# The Effect of a High-Dose Vitamin B Multivitamin Supplement on the Relationship between Brain Metabolism and Blood Biomarkers of Oxidative Stress: A Randomized Control Trial

**DOI:** 10.3390/nu10121860

**Published:** 2018-12-01

**Authors:** Talitha C. Ford, Luke A. Downey, Tamara Simpson, Grace McPhee, Chris Oliver, Con Stough

**Affiliations:** 1 Centre for Human Psychopharmacology, Swinburne University, Melbourne, VIC 3122, Australia; tcford@swin.edu.au (T.C.F.); tsimpson@swin.edu.au (T.S.); gmcphee@swin.edu.au (G.M.); cstough@swin.edu.au (C.S.); 2 Institute for Breathing and Sleep, Austin Hospital, Melbourne, VIC 3084, Australia; 3 Oliver Nutrition, Sydney, NSW 2000, Australia; Christopher.Oliver@scu.edu.au

**Keywords:** B vitamins, multivitamin, 1H-MRS, homocysteine, oxidative stress, posterior cingulate cortex, NAA, creatine

## Abstract

A diet rich in B-group vitamins is essential for optimal body and brain function, and insufficient amounts of such vitamins have been associated with higher levels of neural inflammation and oxidative stress, as marked by increased blood plasma homocysteine. Neural biomarkers of oxidative stress quantified through proton magnetic spectroscopy (1H-MRS) are not well understood, and the relationship between such neural and blood biomarkers is seldom studied. The current study addresses this gap by investigating the direct effect of 6-month high-dose B-group vitamin supplementation on neural and blood biomarkers of metabolism. Using a randomized, double-blind, placebo-controlled design, 32 healthy adults (20 female, 12 male) aged 30–65 years underwent blood tests (vitamin B6, vitamin B12, folate, and homocysteine levels) and 1H-MRS of the posterior cingulate cortex (PCC) and dorsolateral prefrontal cortex (DLPFC) before and after supplementation. Results confirmed the supplement was effective in increasing vitamin B6 and vitamin B12 levels and reducing homocysteine, whereas there was no change in folate levels. There were significant relationships between vitamin B6 and *N*-acetylaspartate (NAA), choline, and creatine, as well as between vitamin B12 and creatine (*p*s < 0.05), whereas NAA in the PCC increased, albeit not significantly (*p* > 0.05). Together these data provide preliminary evidence for the efficacy of high-dose B-group supplementation in reducing oxidative stress and inflammation through increasing oxidative metabolism. It may also promote myelination, cellular metabolism, and energy storage.

## 1. Introduction

The quality of the human diet has a direct effect on body and brain functionality, with several studies pointing toward the efficacy of vitamin and mineral supplementation, particularly B vitamins, in preventing and alleviating disease and disability [1,2,3,4]. Even in healthy humans, multivitamin supplementation has been shown to improve cognitive performance and reduce negative mood states, including depression, anxiety, and stress [5,6,7,8,9,10]. Neuroimaging techniques such as proton magnetic resonance spectroscopy (1H-MRS) have proven effective in quantifying neural markers of metabolism and disease, and can thus be utilised in assessing the efficacy of dietary interventions on such neural markers [8,11,12]. This study is the first to investigate the effect of high-dose B vitamin multivitamin supplementation on neural markers of metabolism, as well as blood biomarkers of inflammation and oxidative stress in healthy adults, allowing for the examination of the relationship between these blood and neural biomarkers as a result of high-dose vitamin B supplementation.

A diet rich in vitamins and micronutrients is essential for optimal body and brain function. B-group vitamins, in particular, are required for various cortical processes involved in metabolism, such as in the methylation of homocysteine to methionine (specifically B6, folate (B9), and B12), which is essential for DNA synthesis, repair, and other methylation reactions in the central nervous system [13,14,15]. Disruption to this methylation process leads to a buildup of homocysteine, which in turn increases the likelihood of cortical inflammation, oxidative stress, and subsequent damage to mitochondria and DNA strands [14,15]. This homocysteine increase, and subsequent neural damage, is linked to deficits in cognitive performance [16].

A deficiency in the B vitamin folate has been implicated in various birth defects, neurodegenerative diseases, and psychiatric conditions [13]. Vitamin B12 deficiency has been associated with several syndromes associated with megaloblastic anaemia and disorders of the nervous system, including numbness and weak and uncoordinated muscles, as well as affective disorders and changes in cognitive performance [2,3]. The symptoms associated with folate and vitamin B12 deficiency are thought to be caused in part by an accumulation of homocysteine, as inadequate availability of these vitamins reduces methionine-homocysteine cycling [13] and leads to reduced myelination [3]. High levels of homocysteine have also been associated with brain atrophy in older adults, through reduced myelination, which is thought to be associated with increased cognitive decline [1]. Dietary supplementation of vitamin B12 and folate have been shown to be effective in alleviating associated syndromes [2,3,4]. In fact, combined vitamin B (folate, B6, and B12) supplementation has been shown to slow the rate of brain atrophy for those with mild cognitive impairment over a 24-month period, which corresponded significantly to increases in B12 and folate levels [1].

In healthy adults, 90 days of high-dose vitamin B multivitamin supplementation has been shown to reduce negative mood states of personal strain, confusion, and depression [7]. More generally, multivitamin supplementation for four weeks has been shown to reduce depressive states [9], stress, anxiety, and tiredness [17], whereas 9-week supplementation reduced fatigue and improved performance during a cognitive task [5], and 16-week supplementation improved Stroop task performance for men but not women [18]. However, the improvement in cognitive performance is task-specific. For example, supplementation improved immediate recall, mental speed, and number facility, but not reasoning, delayed recall, or verbal fluency (for a review, see Reference [10]), and no improvement in reaction time or memory have been reported [18]. Acute multivitamin supplementation has also been shown to improve contentment and cognitive task performance in adults [8]. In children, multivitamin supplementation for 12 weeks has been shown to increase intelligence [19] and cognitive performance, but not mood [6], whereas 4-month supplementation has been shown to have no effect on cognition [20]. These findings together demonstrate that multivitamin supplementation, particularly those higher in B vitamins, may improve cognition and mood, which might be facilitated by improving brain health, although more targeted investigations into the relationship between B vitamins and neuromarkers of health are required.

1H-MRS is a reliable measure of brain markers of metabolism and disease through the quantification of molecules that are involved in metabolism (metabolites) within specific regions of interest, and it is effective in quantifying the low molecular weighted metabolites of *N*-acetylaspartate (NAA), choline-containing compounds (collectively referred to herein as “choline”), creatine (including phosphocreatine), myo-Inositol, and a composite measure of glutamate and glutamine (Glx). Of these readily quantifiable metabolites, NAA is a marker of neural density and viability due to its role in oxidative metabolism and myelination [11,21,22], and may thus be affected by vitamin B supplementation, which has been shown to increase oxidative metabolism and thus reduce neural inflammation and oxidative stress [14]. Choline is involved in cellular membrane metabolism, and thus 1H-MRS-quantified choline concentrations mark cell membrane turnover, cellular growth, proliferation, and breakdown [21,23]. Considering the role of B-group vitamins in methionine-homocysteine cycling, which is essential for cellular metabolism [13], vitamin B supplementation may similarly affect choline concentrations in vivo. Finally, creatine is crucial for cortical homeostasis and cellular energy storage through adenosine triphosphate and adenosine diphosphate cycling, which again interacts with methionine-homocysteine cycling [13,21,24].

Few studies have investigated the relationship between 1H-MRS-quantifiable neural and blood biomarkers of metabolism and neural inflammation. Of those, increased plasma homocysteine has been associated with reduced NAA in the centrum semi-ovale [25] and hippocampus [26], reflecting the neural damage associated with increased homocysteine [25,26]. Increased plasma homocysteine has also been associated with reduced centrum semi-ovale creatine concentrations, suggesting that neural inflammation leads to a reduction in energy metabolism [25]. No significant relationships have been detected between homocysteine levels and metabolites in the left frontal lobe or basal ganglia [26], or choline in the centrum semi-ovale [25], suggesting that the relationship between homocysteine and metabolite concentrations may be regionally specific.

Of the studies investigating the relationship between blood and brain biomarkers, none have investigated brain biomarkers in the highly connected region of the posterior cingulate cortex (PCC). The PCC is particularly highly connected throughout the cortex, playing a central role in the default mode network, as well as in focus and attention [27]. As such, the PCC is highly metabolically active, even at rest, with a metabolic rate around 40% higher than other cortical regions on average [28]. Considering the metabolic load of the PCC, and the role of vitamin B in metabolic processes, investigating the effect of supplementation on metabolite concentrations within the PCC might shed some light on the utility of 1H-MRS in measuring the underlying metabolic mechanisms associated with nutrition and brain function [11]. Similarly, no studies have investigated the relationship between blood biomarkers and metabolite concentrations in the dorsolateral prefrontal cortex (DLPFC), a central hub for cognition and executive functions. The DLPFC is also highly metabolically demanding, especially during cognitive tasks [29]. In addition to there being no studies investigating the relationship between blood biomarkers of metabolism and cortical metabolites in the PCC or DLPFC, the direct effect of high-dose B vitamin multivitamin supplementation on blood biomarkers of metabolism and metabolite concentrations concurrently is yet to be examined.

This study is the first to investigate the direct effect of 6-month high-dose B vitamin multivitamin supplementation on 1H-MRS metabolite and blood biomarkers, and the relationships between these biomarkers, in healthy adults. It was hypothesized that 6-month vitamin B supplementation would increase blood plasma vitamin B6, vitamin B12, and red blood cell folate levels, and reduce blood plasma homocysteine levels, which would lead to increased NAA, choline, and creatine concentrations. Furthermore, it was predicted that increased homocysteine would be associated with reduced NAA, choline, and creatine concentrations in the PCC and DLPFC.

## 2. Materials and Methods

These data were collected as part of a larger (*n* = 200) randomised, placebo-controlled, double-blind parallel groups design investigating the effects of Blackmores^®^ Executive B Stress Formula on the primary outcome of work-related stress, as well as on a range of secondary cognitive, stress, mood, health, personality, cardiovascular, biochemical, genetic, and neuroimaging outcomes [30]. The 1H-MRS data from the smaller neuroimaging sub-study (*n* = 39) are the focus of this paper. The study was registered with the Australian and New Zealand Clinical Trials Registry (ACTRN12613000294752), and was approved by the Swinburne University Research Ethics Committee (SUHREC 2012/293). All participants provided written informed consent to participate in the study.

### 2.1. Participants

Of the 39 adults aged 30 to 65 years (25 female, 14 male) enrolled in the neuroimaging sub-study, a total of 36 underwent 1H-MRS at baseline (female, male), and 33 participants at 6 months (21 female, 12 male). See Table 1 for characteristics of the final sample and Figure S1 for participant enrollment flow chart. Participants in the 1H-MRS sub-study were enrolled starting in February 2014, with testing ceasing in December 2016. Participants were non-smokers, not heavy consumers of alcohol (i.e., females consumed <14 standard drinks per week, whereas males consumed <28 standard drinks per week), not current or past illicit drug users, and did not have a history of psychiatric illness (history of anxiety, depression, psychiatric disorders), epilepsy, or a neurological condition. A medical screening was conducted to confirm the above criteria and to confirm the absence of clinically relevant abnormalities in their medical history that would render them ineligible for Magnetic Resonance Imaging (MRI) (e.g., claustrophobia, metal implant, cardiac pacemaker or defibrillator, spinal cord stimulator, or pregnancy). Participants were asked to refrain from drinking alcohol for 24 h prior to the scheduled study day, and to not drink caffeine 12 h prior to the study time.

### 2.2. Procedure

Participants attended the Centre for Human Psychopharmacology at Swinburne University for three separate sessions. Following telephone screening for initial eligibility, participants attended a screening session in which eligibility was confirmed by the investigators. Eligible participants were randomized into placebo (*n* = 20) or active (*n* = 19) treatment conditions by random allocation using a computerized random number generator by a disinterested third party, ensuring all investigators remained blind to the treatment allocation. Participants then attended a pretreatment (baseline) testing session and a post-treatment testing session 6 months later. At the end of the screening session, enrolled participants were provided their assigned treatment and administration instructions as detailed in the “Treatment” section below.

At the baseline and 6-month testing sessions, participants completed a food frequency questionnaire, provided fasted blood samples for plasma vitamin B12, vitamin B6, homocysteine, and red blood cell folate, and underwent 1H-MRS to quantify low molecular weighted metabolites: NAA, creatine, and choline in the PCC and left DLPFC. Biochemical and 1H-MRS data collection methods are detailed in data collection sections below. Participants were contacted monthly to record any significant life or diet events that may have occurred and to check that they were taking tablets daily. The remaining tablets were counted at the 6-month testing session to ensure compliance with the treatment schedule.

### 2.3. Treatment

Active and placebo treatments were in the form of large dark brown film-coated cream tablets, matched in color and size, and were manufactured by Blackmores^®^ Australia. The nutrients doses of each tablet are detailed in Table 2, as well as the nutrients’ percentage of recommended daily intake according to the National Health and Medical Research Council of Australia [31]. All B vitamin doses were well above the recommended daily intake for adults aged 30–70 years, except biotin (vitamin B7, 66.7–80%) and folic acid (vitamin B9, 37.5%), confirming the supplement contained a high dose of B vitamins overall.

Participants were provided the treatment in unmarked bottles and instructed to take two tablets daily, one at breakfast and one at lunch, for the duration of 6 months. Each participant received enough tablets for 6 months, along with an additional week of tablets in case the post-treatment visit date was delayed, resulting in 350 tablets in total. To prevent any acute supplementation effects, participants were asked not to take any tablets on the day of their post-treatment testing session. Blackmores^®^ Executive B Stress Formula is available over the counter. The placebo tablets contained a small amount of glucose and riboflavin (B2, 2 mg) to be matched for colour and taste and to provide a similar urine coloration effect. No adverse events were reported as a result of the active or placebo treatment, or as a result of the study procedures.

### 2.4. Blood Biomarker Data Collection

Blood sampling was conducted via venepuncture on each of the testing days following a period of fasting from the night before at the testing site by a registered nurse. Blood biomarkers of vitamin B12, vitamin B6, folate, and homocysteine were obtained. Additionally, safety profiling was measured through a full blood count, high-sensitivity C-reactive protein, and biochemical liver function tests.

### 2.5. 1H-MRS Biomarker Data Collection

All 1H-MRS and T1-weighted images were recorded from a 3T Siemens TIM Trio whole-body MRI system (Erlangen, Germany) with a 32-channel head coil at the Swinburne University Neuroimaging Facility. T1-weighted images, for a 1H-MRS voxel of interest localization and tissue composition, were acquired sagittally using a magnetization prepared rapid gradient echo (MPRage) pulse sequence with an inversion recovery (176 slices, slice thickness = 1.0 mm, voxel resolution = 1.0 mm^3^, TR = 1900 ms, TE = 2.52 ms, TI = 900 ms, bandwidth = 170 Hz/Px, flip angle = 9°, field of view 350 × 263 × 350 mm, orientation sagittal, acquisition time = 5 min).

The T1 image was used to position the PCC (20 × 20 × 20 mm) and left DLPFC (15 × 20 × 20 mm) voxels (Figure S2). For localised quantification of total NAA (NAA + NAA-glutamate), total creatine (creatine + phosphocreatine), and total choline (phosphocholine + glycerylphosphorylcholine), a PRESS sequence was employed with chemical shift selective (CHESS) [32] water suppression (TE = 30 ms, TR = 2000 ms, bandwidth = 1200 Hz, 80 averages for PCC, 160 averages for DLPFC, acquisition time = 2 min 48 s). Eight and 16 spectral water averages (without water suppression) were acquired with identical PRESS parameters and shim for the PCC and left DLPFC voxels, respectively.

### 2.6. 1H-MRS Biomarker Data Analysis

Analyses were conducted with TARQUIN version 4.3.7, which estimates signal amplitude using a non-negative least-squares projection of a parametrised basis set in the time domain [33]. Eddy current correction was applied. For both PCC and DLPFC voxels, data were excluded if (a) the signal-to-noise ratio (SNR) was less than 10, (b) the water line width (FWHM_water_) was greater than 12 Hz, or (c) the visual inspection failed. Two female participants’ PCC data were excluded from statistical analyses (FWHM_water_ > 12 Hz), and one female participant’s data were excluded due to being an extreme value (placebo *n* = 2, active *n* = 1). There were no group differences in SNR, FWHM_water_, grey matter, white matter, and cerebrospinal fluid (CSF, see Table 3 for final sample fit statistics). Of the DLPFC data, over half of the spectra did not meet fit inclusion criteria, and therefore all DLPFC data were excluded from further analyses. All metabolite concentrations were corrected for voxel CSF ratio and water concentration (due to water scaling) using the following formula [34]:

*M_corr_* = *M* ∗ *(WM* + *GM* + 1.55 × *CSF)*/*(WM* + *GM)*, (1)

where *M_corr_* is the corrected metabolite concentration, *M* is the original metabolite concentration, and *WM* and *GM* are white matter and grey matter percentages, respectively.

### 2.7. Statistical Analysis

Based on estimates from a 12-week intervention of an omega-3 supplement on brain metabolites [35], power analysis suggested that for fixed effects mixed linear models to detect a large effect (*f*^2^ = 0.5, α = 0.05, power = 0.8, 2 groups), 34 participants were required (G*Power version 3.1: http://www.gpower.hhu.de/, Heinrich-Heine-Universität Düsseldorf, Germany). A series of linear mixed effects analyses with restricted maximum likelihood were conducted to investigate the effect of the treatment on blood biomarker and brain metabolites concentrations in the PCC. Treatment group (active vs. placebo), time (baseline vs. 6 months), and their interaction were entered as fixed effects, and the intercept for subject was entered as a random effect to account for within-subject variability. Although there were no significant group differences in age (*p* > 0.1), age was entered as a covariate given its effect on brain metabolite concentrations. There were no differences in dietary intake across food groups between the groups (*p*s > 0.1).

Spearman’s rho (ρ) rank order correlation coefficients probed the relationship between blood biomarkers and metabolite concentrations in the PCC. This method is less sensitive to univariate outliers and non-normal data [36]. A *p*-value of <0.05 was deemed significant. All group by time interactions and Spearman’s rank order correlation *p*-values were adjusted for false discovery rate (FDR, *q*) using the fdrtool in R [37]. All analyses were conducted using jamovi and the GAMLj package [38].

## 3. Results

### 3.1. Blood Biomarker Group Differences

Separate mixed linear models were conducted to test the effect of the treatment group (active vs. placebo) and time (baseline vs. 6 months) on blood plasma concentrations of homocysteine, vitamin B6, vitamin B12, and red blood cell folate. Means and standard deviations by treatment group and time are presented in Table 4. The treatment group by time interaction terms for vitamin B6 and vitamin B12 concentrations were significant after controlling for FDR (*q*), suggesting that in both cases, biomarker levels increased as a result of the treatment (vitamin B6: *F*[1, 27.8] = 23.66, *b* = 117.31, 95% CI [70.04, 164.58], *p* < 0.001, *q* < 0.001; vitamin B12: *F*[1, 25.27] = 16.35, *b* = 42.74, 95% CI [22.02, 63.46], *p* < 0.001, *q* < 0.001). There was also a significant group by time interaction for plasma homocysteine concentration, with the treatment resulting in reduced homocysteine (*F*[1, 26.22] = 27.52, *b* = −0.68, 95% CI [−0.93, −0.42], *p* < 0.001, *q* < 0.001). There was no significant group by time interaction for red blood cell folate, suggesting that folate levels did not change as a result of treatment (*F*[1, 26.23] = 0.35, *b* = −12.77, 95% CI [−55.05, 29.51], *p* = 0.559).

### 3.2. 1H-MRS Metabolite Biomarker Group Differences

**Figure 1:**
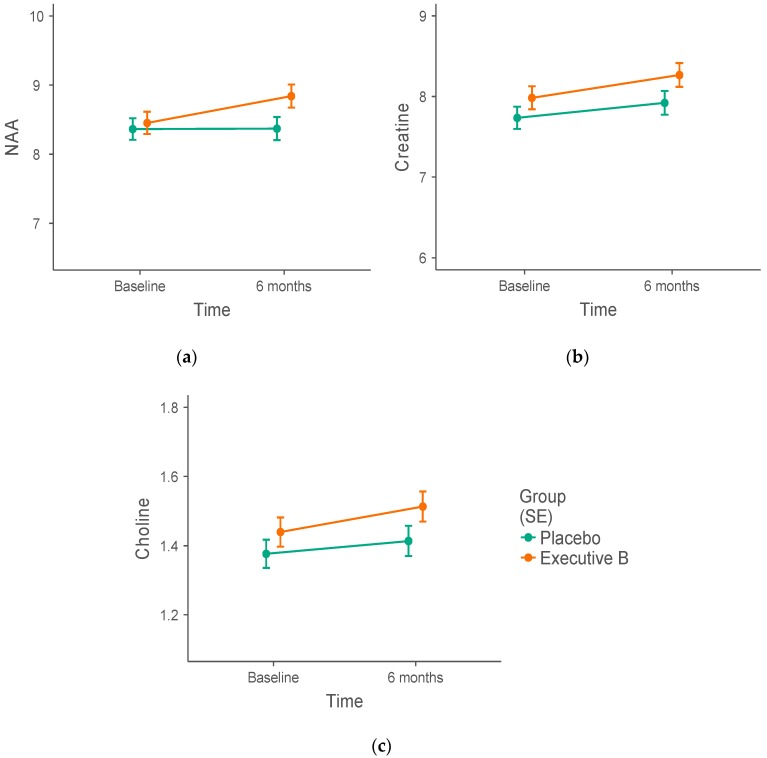
*N*-acetylaspartate (NAA), choline, and creatine concentrations in the posterior cingulate cortex (PCC). Effect of 6-month high-dose B vitamin multivitamin (Executive B) supplementation on (**a**) NAA, (**b**) creatine, and (**c**) choline concentrations in the PCC. Despite visual differences between the Executive B active and placebo intervention groups, the treatment group by time interaction was not significant (NAA: *b* = 0.10, 95% CI [−0.04, 0.24], *p* = 0.194).

Separate mixed linear models were conducted to test the effect of the treatment group (active vs. placebo) and time (baseline vs. 6 months) on metabolite concentrations in the PCC (NAA, choline, and creatine). Means and standard errors by group and time are presented in Table 4. There were no significant group by time interactions (*p* > 0.05, Figure 1), suggesting no effect of the treatment on any of the metabolite concentrations in the PCC. Visual inspection of the concentrations suggests that the treatment intervention led to an increase in PCC NAA concentration (Figure 1a). However, this increase was not statistically significant (*F* [1, 26.07] = 1.78, *b* = 0.10, 95% CI [−0.04, 0.24], *p* = 0.194).

### 3.3. Blood and Metabolite Biomarker Correlations

Spearman rank order correlations (ρ) revealed no significant relationships between blood and metabolite biomarker concentrations in the PCC at baseline (*p*s > 0.05). In contrast, at 6 months post-treatment there were significant, positive correlations between vitamin B6 levels and NAA (ρ = 0.523, *p* = 0.007), creatine (ρ = 0.413, *p* = 0.040), and choline (ρ = 0.489, *p* = 0.013) concentrations; and vitamin B12 and creatine concentrations (ρ = 0.406, *p* = 0.041), whereas there was marginal significance for choline concentration (ρ = 0.359, *p* = 0.073). There were no other significant correlations between blood biomarkers and metabolite concentrations at 6 months.

Spearman rank order correlations between the blood biomarkers revealed significant negative correlations between post-treatment homocysteine and vitamin B6 (ρ = −0.479, *p* = 0.013), vitamin B12 (ρ = −0.397, *p* = 0.040), and folate (ρ = −0.453, *p* = 0.018). After controlling for FDR, however, none of the abovementioned correlations were significant (*q* > 0.05).

## 4. Discussion

This study is the first to investigate the direct effect of 6-month high-dose B vitamin multivitamin supplementation on 1H-MRS metabolite and blood biomarkers, and the relationships between these biomarkers, in healthy adults. Following the 6-month supplementation, the expected increase in blood plasma vitamin B6 and B12 was observed, as well as a reduction in blood plasma homocysteine levels. Brain metabolite concentrations in the PCC, a region that is highly connected across the cortex and is highly metabolically active [27], were not significantly affected by the intervention, although there was preliminary evidence for increased NAA concentration, which appeared to be associated with increased blood plasma vitamin B6 levels. Post-intervention vitamin B6 levels were also associated with increased choline and creatine, whereas increased vitamin B12 was associated with increased creatine concentrations. Together, these findings provide preliminary evidence for the utility of vitamin B supplementation in reducing inflammation and oxidative stress, and in promoting neural metabolic processes.

Although vitamin B6, vitamin B12, and folate share a role in maintaining cardiovascular, neural, and psychological health [2,3,4] through the regulation of homocysteine [13,14,15], these data suggest that the increase in plasma vitamin B6 and vitamin B12 drove the reduction in plasma homocysteine levels in this study, given there was no significant change in folate levels. The catalysis of homocysteine is driven by vitamin B6- and vitamin B12-dependent enzymes (cystathionine B-synthase and methionine synthase, respectively) [13], suggesting that supplementation with a high-dose B vitamin multivitamin may promote the breakdown of homocysteine to a greater extent than folate. In Australia, however, many food products are fortified with folate (e.g., bread and milk), which might explain the minimal change in red cell folate levels. This absence of folate level change has been previously reported following 4-week supplementation of a multivitamin in an Australian sample, despite increased vitamin B6 and B12 and decreased homocysteine [9].

The relationship between blood and brain metabolic processes was investigated with 1H-MRS, as the brain metabolites NAA, creatine, and choline are markers of cellular membrane, energy, and oxidative metabolism, which involve the blood biomarkers homocysteine, vitamin B6, vitamin B12, and folate [11,12]. Despite this known interaction, few studies have formally investigated the relationship between brain and blood biomarkers of metabolism. These data demonstrated that, as expected, increases in plasma vitamin B6 were associated with increases in brain NAA, creatine, and choline in the PCC, a region that is highly connected across the cortex and is highly metabolically active [27]. Furthermore, there was preliminary evidence for an increase in PCC NAA concentrations following the 6-month supplementation. NAA is involved in oxidative metabolism and myelination, and NAA concentrations quantified using 1H-MRS are thought to mark neural density and viability [11,21,22]. This is further evidenced by reduced NAA following neurological injury, which suggests demyelination and reduced oxidative metabolism and has been implicated in cognitive deficits and pathology (for a review, see Reference [11]).

There was, however, no relationship between vitamin B12 or folate and NAA concentrations, suggesting that the metabolic pathway responsible for catalyzing homocysteine to cysteine, to which vitamin B6 is a co-factor [39], might be more closely related to NAA concentrations. Similarly, choline concentrations were associated with increased vitamin B6 levels only, suggesting that cell membrane turnover (cellular growth, proliferation, and breakdown) may be more directly related to the removal of homocysteine completely, rather than the regulatory demethylation-remethylation cycle that depends on vitamin B12 and folate. Thus, 1H-MRS may be a valuable tool with which to quantify markers of the breakdown of homocysteine, oxidative metabolism, and cell membrane turnover in brain regions that are specialized to serve particular functions, providing valuable information regarding the role of regional oxidative metabolism and cell membrane turnover in both healthy and diseased populations.

Finally, higher plasma vitamin B6 and B12 levels were related to increased creatine concentrations in the PCC, which was predicted given that 1H-MRS-quantified creatine marks cellular energy metabolism, energy storage, and cortical homeostasis through its crucial role in adenosine triphosphate and adenosine diphosphate energy transfer [21,24]. Vitamin B6 and B12 are involved in homocysteine metabolism through remethylating homocysteine to methionine and catalyzing homocysteine to cysteine, respectively. Together, therefore, these vitamins are essential in regulating oxidative metabolism and thus protecting against oxidative stress [14,15]. The finding of increased vitamin B6 and B12 levels with higher creatine concentrations suggests that supplementation contributes to increased cellular metabolism in the PCC, and that creatine quantified with 1H-MRS might be a viable marker for both aspects of homocysteine metabolism.

A few limitations of this study require mentioning. First, the small sample size led to several subthreshold group differences and relationships. These results should thus be interpreted as preliminary findings that warrant further investigation with a larger sample. Second, the intervention timeframe of 6 months may have been too short for significant changes in neural biochemical markers of metabolism to occur in a healthy sample, thereby limiting the ability to detect intervention-related metabolite changes. Third, fortification of folate in many Australian foods may have reduced the effect of the supplement on red blood cell folate levels in this sample and thus impacted any subsequent significant changes in metabolite levels. It should be noted, therefore, that the supplement may have greater impact on folate for those who do not consume fortified food products. There were, however, no differences between the groups in dietary factors. Fourth, this study focused specifically on the efficacy of the high B vitamin multivitamin on blood markers of oxidative stress (vitamin B6 and B12, folate, and homocysteine). However, it should be acknowledged that additional blood-vitamin concentration changes due to the supplement (e.g., B1, B5, B7) may have moderated some of the results of this study. Furthermore, the supplement contained a number of additional vitamins and minerals that may have affected the relationship between blood and brain markers. Given there were no between-group metabolite differences, it is unlikely that the additional vitamins affected the relationships revealed between the blood and brain markers. Finally, the majority of the DLPFC data were unusable due to poor linewidths and signal to noise, ultimately limiting our ability to investigate the relationship between metabolites in this executive functioning-specific brain region and the blood biomarkers. Nevertheless, as a preliminary study investigating the relationship between PCC neural and blood biomarkers, and the effect of vitamin B supplementation on this relationship, the findings of the current intervention study provide important insight into the utility of different modalities to investigate body and brain health.

## 5. Conclusions

This study was the first to investigate the efficacy of high-dose B vitamin multivitamin supplementation in modulating the relationship between neural and blood biomarkers of oxidative stress. Blackmore’s^®^ Executive B Stress Formula was shown to reduce blood markers for oxidative stress (homocysteine) and increase brain markers for oxidative metabolism and myelination, but not energy or cellular membrane metabolism. Increasing levels of blood high-dose B-group vitamins were also associated with increased neural metabolism. These findings suggest that high-dose B-group vitamin supplementation might be effective in reducing oxidative stress and inflammation through increasing oxidative metabolism, and may promote myelination, cellular metabolism, and energy storage. Together, these findings highlight the importance of B-group vitamins in the maintenance of brain health in healthy adults and may have important implications in the prevention and alleviation of disease and disability.

## Figures and Tables

**Table 1:** Demographic information of final sample.

		Baseline		6 Months	
		Active	Placebo	Active	Placebo
Sex	Female	9	11	8	10
	Male	6	6	6	4
	Total	15	17	14	14
Age	30–38	5	6	5	5
	39–47	6	4	5	3
	48–56	2	4	2	3
	57–65	2	3	2	3
	Total	15	17	14	14

**Table 2:** Ingredients and doses of Blackmores^®^ Executive B Formula.

Active Ingredients (per tablet)	Dosage	RDI/AI	
		Male	Female
Vitamin B1 (thiamine)	75 mg	6250%	6818%
Vitamin B2 (riboflavin)	10 mg	769%	909%
Vitamin B3 (nicotinamide/niacin)	100 mg	625%	714%
Vitamin B5	68.7 mg	1145%	1718%
Vitamin B6 (pyridoxine)	25 mg	1923% *	1923% *
		1470% ^#^	1667% ^#^
Vitamin B7 (biotin)	20 μg	66.7%	80%
Vitamin B9 (folic acid)	150 μg	37.5%	37.5%
Vitamin B12 (cyanocobalamin)	30 μg	1250%	1250%
Calcium phosphate	100 mg	10%	10%
Calcium ascorbate	145 mg	14.5%	14.5% *
			11.2% ^#^
Ascorbic acid	130 mg	289%	289%
Total vitamin C	250 mg	556%	556%
d-alpha-tocopheryl acid succinate (vitamin E)	41.3 mg	413%	590%
Magnesium phosphate	140 mg	33.3%	43.8%
Potassium phosphate monobasic	117.3 mg	3.1%	4.1%
Choline birartrate	25 mg	4.5%	5.9%
Lecithin	50 mg	NR	NR
Inositol	25 mg	NR	NR
Avena sativa (oats)	100 mg	NR	NR
Passifloraincarnata (passion flower)	250 mg	NR	NR

Ingredient dosage and micronutrient Recommended Daily Intake (RDI) or Adequate Intake (AI) according to the Nutrient Reference Values for Australia and New Zealand [31]. Recommendations are for the age range of 30–70 years, * = 31–50 years, ^#^ = 51–70 years. NR = not reported.

**Table 3:** Means and standard deviations for 1H-MRS fit statistics of the posterior cingulate cortex voxel data between groups.

	Baseline		6 Months	
	Active	Placebo	Active	Placebo
*N*	15	15	13	13
SNR	22.96 (3.68)	26.38 (3.56)	24.91 (2.37)	26.92 (4.29)
FWHM_water_	8.15 (1.38)	8.38 (1.35)	7.84 (0.88)	7.91 (1.05)
GM (%)	73.27 (3.59)	72.39 (4.35)	72.36 (3.73)	71.81 (3.80)
WM (%)	22.17 (3.48)	23.55 (4.89)	22.83 (3.43)	23.57 (4.58)
CSF (%)	4.56 (1.58)	4.05 (1.63)	4.82 (1.82)	4.63 (1.79)

SNR = signal to noise ratio, FWHM_water_ = full-width half-maximum for the water peak, GM = grey matter, WM = white matter, CSF = cerebrospinal fluid.

**Table 4:** Means (M) and standard error (SE) for blood and metabolite biomarker concentrations between groups.

		Baseline		6 Months	
		Active	Placebo	Active	Placebo
Blood	*N*	15	15	14	13
	HCy (μmol/L)	9.57 ± 0.49	8.95 ± 0.51	7.98 ± 0.46 *	10.05 ± 0.54
	Vit B6 (nmol/L)	191.07 ± 68.2	124.13 ± 34.6	619.79 ± 76.4 *	82.25 ± 5.6
	Vit B12 (pmol/L)	285.53 ± 21.5	296.27 ± 28.4	443.50 ± 43.3 *	275.85 ± 25.8
	Folate (nmol/L)	1265.60 ± 58.8	1141.27 ± 57.9	1317.07 ± 45.7	1242.08 ± 42.9
1H-MRS	*N*	14	15	13	13
PCC	NAA (IU)	8.46 ± 0.17	8.36 ± 0.20	8.85 ± 0.13	8.38 ± 0.14
	Choline (IU)	1.44 ± 0.05	1.38 ± 0.04	1.52 ± 0.03	1.42 ± 0.04
	Creatine (IU)	7.97 ± 0.19	7.74 ± 0.15	8.23 ± 0.12	7.96 ± 0.13
	mI (IU)	4.95 ± 0.21	4.53 ± 0.15	4.99 ± 0.13	4.73 ± 0.15
	Glx (IU)	13.56 ± 0.70	11.44 ± 0.47	12.05 ± 0.37	11.82 ± 0.64

Note: HCy = homocysteine, Vit = vitamin, PCC = posterior cingulate cortex, mI = myo-Inositol, Glx = glutamate + glutamine, IU = institutional units. Data presented as M ± SE; * = *p* < 0.001.

## Supplementary Materials

The following are available online at http://www.mdpi.com/2072-6643/10/12/1860/s1, Figure S1: Recruitment Flowchart, Figure S2: 1H-MRS Voxel Placement.
