# Supplementary figures and images for: The Effect of a High-Dose Vitamin B Multivitamin Supplement on the Relationship between Brain Metabolism and Blood Biomarkers of Oxidative Stress: A Randomized Control Trial
Source: Nutrients. 2018 Dec 1;10(12):1860. doi: 10.3390/nu10121860 (PMC6316433; doi:10.3390/nu10121860)

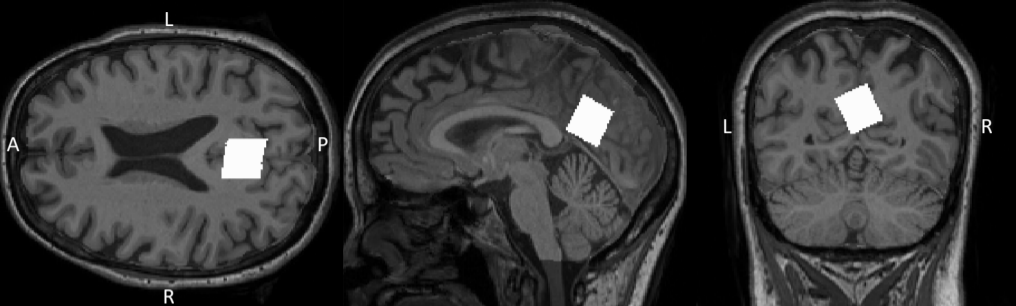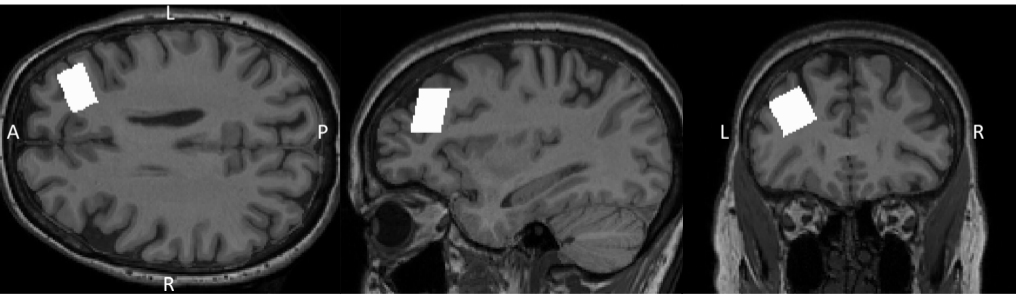

Supplement: Supplementary file 1 [file nutrients-10-01860-s001.zip › Figure_S2.pdf]

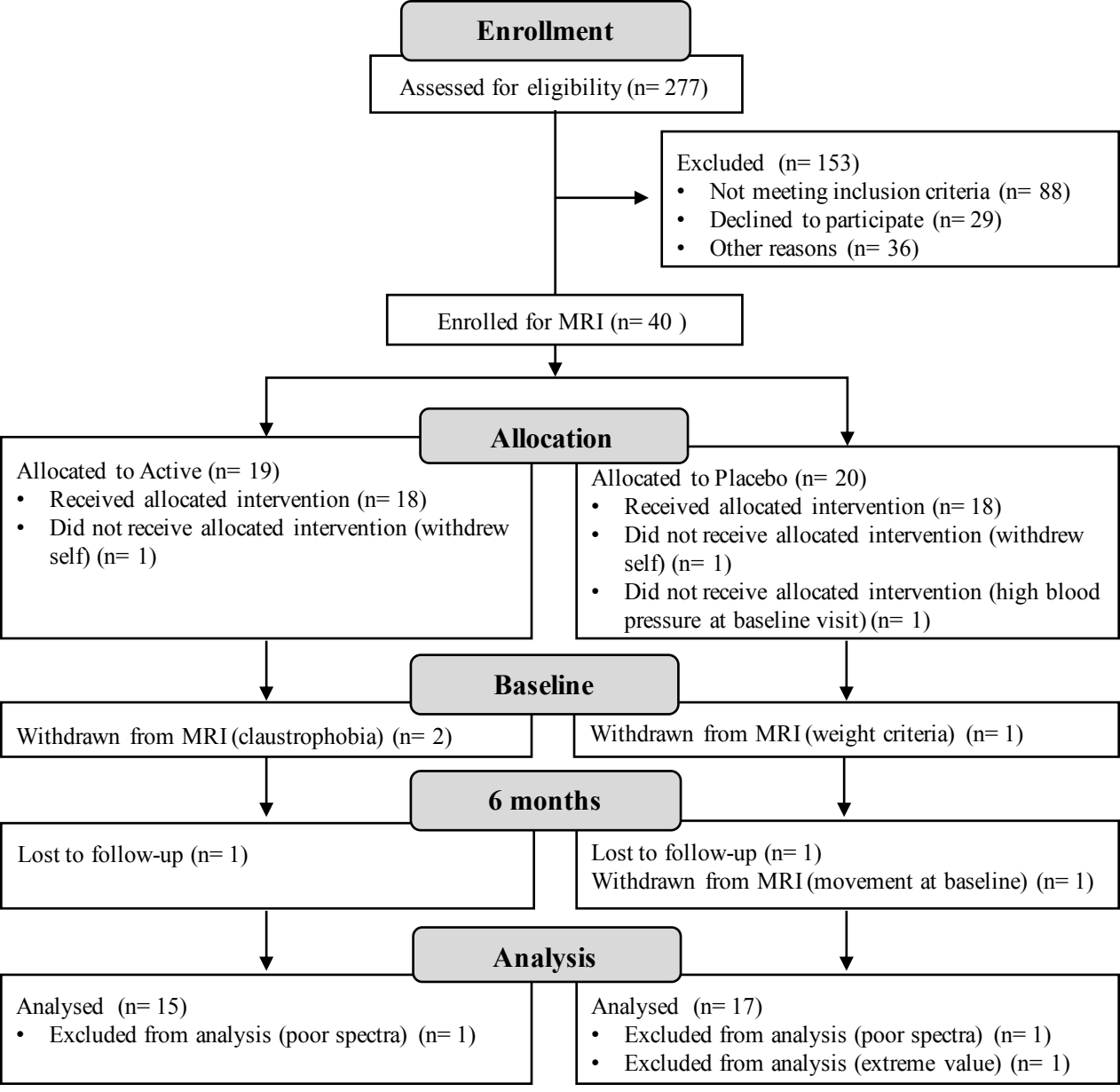

Supplement: Supplementary file 1 [file nutrients-10-01860-s001.zip › Figure_S1.pdf]
